# Comparing ultrasound-derived fat fraction and CT for diagnosing hepatic steatosis: an MRI-PDFF reference study

**DOI:** 10.3389/fmed.2026.1799359

**Published:** 2026-06-10

**Authors:** Huizhen Yu, Yi Li, Weiwen Cai, Yuli Liu, Ying Liu, Hong Wen, Guo Li, Hong Zhou, Jin Gao, Yang Zhou

**Affiliations:** 1 Department of Ultrasound, The Third People's Hospital of Chengdu, Chengdu, Sichuan, China; 2 Department of Radiology, The Third People's Hospital of Chengdu, Chengdu, Sichuan, China

**Keywords:** chronic liver disease, CT liver-to-spleen ratio, hepatic steatosis, MRI-PDFF, UDFF

## Abstract

**Objectives:**

This study directly compares the diagnostic performance of two routinely available, non-invasive techniques—ultrasound-derived fat fraction (UDFF) and non-contrast computed tomography (NCCT)—for screening and grading hepatic steatosis (HS). In addition, it exploratorily assesses the utility of liver stiffness measurement (LSM) for quantifying liver fat content.

**Methods:**

In this prospective single-center study, 78 patients with chronic liver disease (CLD) were recruited and underwent NCCT, ultrasound [UDFF and Automatic Point Shear Wave Elastography (Auto-pSWE)], and magnetic resonance imaging-proton density fat fraction (MRI-PDFF) within 1 week. The same cohort was analyzed using two different MRI-PDFF threshold criteria: the primary set and the alternative set. In the primary set, Spearman's correlation coefficients assessed relationships among UDFF, CT liver-to-spleen attenuation ratio [CT(L/S)], LSM, and MRI-PDFF. The diagnostic performance of UDFF and CT(L/S) for grading HS was evaluated using ROC curves with bootstrap AUC comparisons. Agreement and reliability were assessed using ICC and kappa statistics, and influencing factors were examined by logistic regression. In the alternative set, the diagnostic performance of UDFF and CT(L/S) for grading steatosis was similarly evaluated using ROC curves with bootstrap AUC comparisons.

**Results:**

After excluding three patients, 75 were included in the final analysis. In the primary set, for HS ≥ S1, UDFF (AUC = 0.926; cutoff 8.5%) outperformed CT(L/S) (AUC = 0.826; cutoff 0.95; *p* < 0.05). For HS ≥ S2, both methods performed similarly [UDFF AUC = 0.961; CT(L/S) AUC = 0.943; *p* > 0.05]. Grading diagnosis agreement was good between UDFF and MRI-PDFF (κ = 0.740), but only moderate for CT(L/S) (κ = 0.517). UDFF demonstrated higher inter-operator (ICC = 0.949) and intra-operator (ICC = 0.967) reliability than CT(L/S). Triglyceride level was the determinant factor for UDFF (OR = 8.510, *p* < 0.05). In the Alternative Set, UDFF also outperformed CT(L/S) for HS ≥ S1 (*p* < 0.05), with no significant difference for HS ≥ S2 (*p* > 0.05).

**Conclusion:**

UDFF outperforms CT(L/S) in steatosis grading and reliability but is limited to the Siemens DAX probe, whereas CT(L/S) is equipment-independent but less reliable.

## Highlights

- Both non-contrast CT and ultrasound are routinely available, non-invasive techniques that enable opportunistic screening and grading of hepatic steatosis in patients with chronic liver disease.
- UDFF demonstrates higher accuracy and reliability compared to CT(L/S) and shows better diagnostic agreement in the grading of HS.

## 1 Introduction

Chronic liver disease (CLD) is a progressive condition marked by hepatocyte injury, inflammation, and fibrosis caused by various factors including chronic viral hepatitis B (CHB), metabolic dysfunction-associated steatotic liver disease (MASLD, formerly NAFLD), and alcohol-related liver disease (ALD), which may also co-occur. MASLD represents the most common form of CLD, with a global prevalence of approximately 32.4% and a rate as high as 44.4% in China (1, 2). Hepatic steatosis (HS) is a central feature of CLD that not only contributes to hepatocellular injury but also accelerates progression to fibrosis, cirrhosis, and hepatocellular carcinoma (3, 4). The early diagnosis and grading of HS facilitate timely clinical intervention and stratified management. Therefore, accurate, reproducible, and accessible non-invasive methods are needed.

Liver biopsy remains the diagnostic gold standard for HS, it is limited by its invasiveness, poorly tolerated, and subject to sampling errors (5). While magnetic resonance imaging-proton density fat fraction (MRI-PDFF) serves as the non-invasive reference standard, its high cost and incompatibility with metallic implants or claustrophobia hinder routine use (6). In contrast, non-contrast computed tomography (NCCT) is widely available and frequently detects HS incidentally during scans for other indications, such as emergency chest and abdomen exams or cancer screening (7–9). Liver fat quantification on NCCT uses attenuation-based metrics including liver attenuation, liver-spleen attenuation difference, and the liver-to-spleen attenuation ratio (CT[L/S]) (10, 11). Among these, CT(L/S) is considered the most robust as it is less affected by systemic disease or scanner variability. However, its diagnostic accuracy, particularly for mild HS, remains controversial (12).

Quantitative ultrasound (QUS) has been widely used for HS evaluation due to its safety, accessibility (13). A newer QUS technique, ultrasound-derived fat fraction (UDFF), quantifies liver fat content by analyzing both attenuation coefficient and backscatter coefficient (14). Prior studies have shown strong correlations between UDFF and MRI-PDFF or histologic fat content (15, 16). However, the relationship between HS and liver stiffness measurement (LSM) is unclear, with conflicting evidence on whether fat accumulation affects liver stiffness (17, 18).

Currently, no study has directly compared UDFF with NCCT for HS assessment, and few studies have used LSM for quantitative assessment of HS. Therefore, this prospective study aims to compare the diagnostic performance of UDFF, LSM, and CT(L/S) for HS, using MRI-PDFF as the reference standard.

## 2 Methods

### 2.1 Research approval and participants.

The study was conducted in accordance the 1964 Helsinki Declaration and approved by the Institutional Review Board ([2025] S-110). Written informed consent was obtained from all participants.

This prospective single-center study recruited 78 participants between April and December 2025. Inclusion criteria: (1) age ≥ 18 years; (2) clinically diagnosed CLD (detailed in Supplementary material, Part 1); (3) indications for NCCT (chest/abdomen) and abdominal ultrasound; (4) provided consent. Exclusion criteria: (1) other liver conditions (e.g., decompensated cirrhosis, hepatocellular carcinoma), malignancy-associated cachexia, and cardiopulmonary insufficiency; (2) non-homogeneous fatty liver; (3) splenectomy; (4) MRI contraindications; (5) incomplete or >1-week interval between imaging studies. Three patients were excluded (one splenectomy, two timing violations), yielding 75 patients for final analysis.

**Figure 1:**
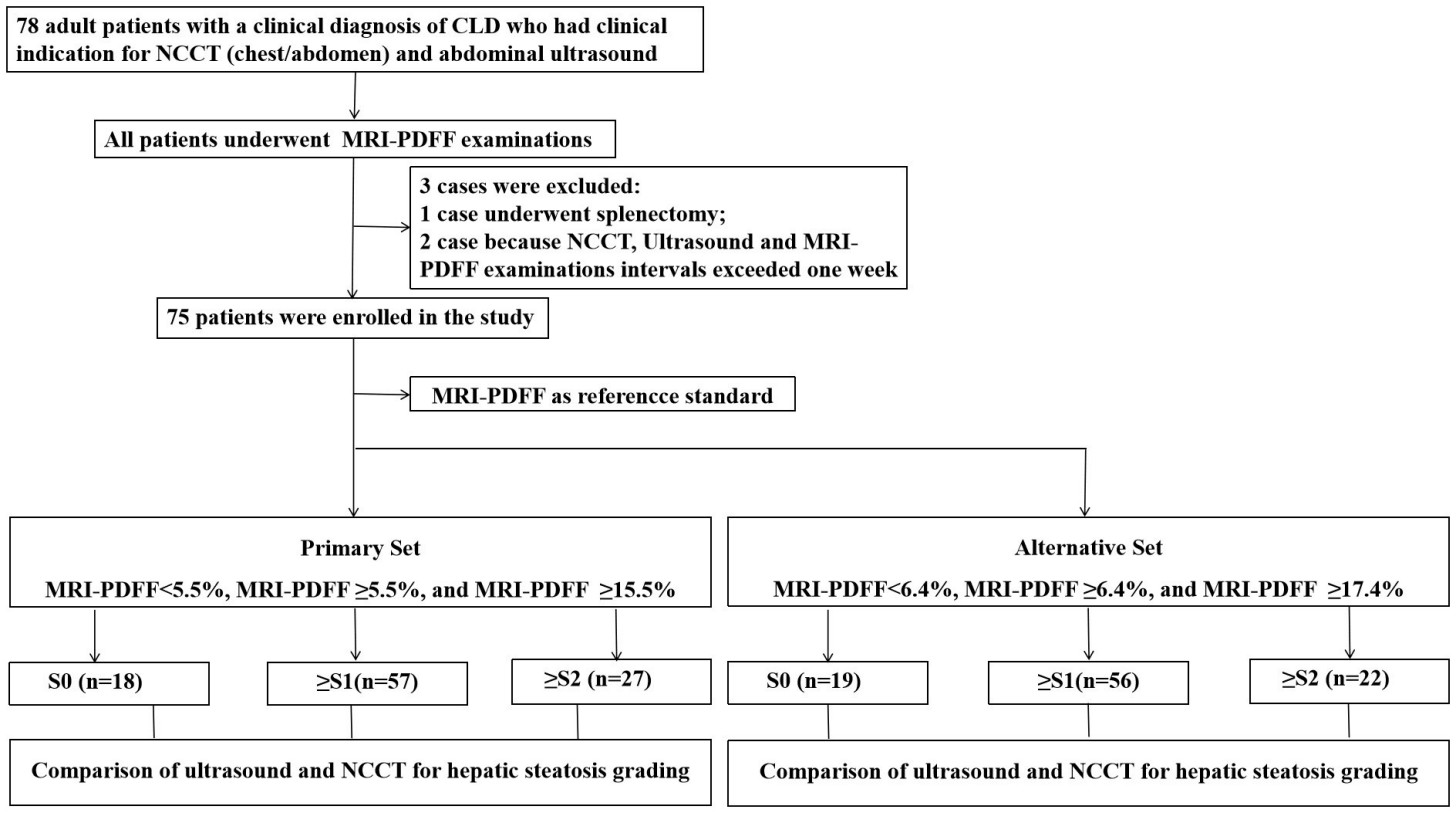
Study flowchart. CLD, chronic liver disease; NCCT, non-contrast computed tomography; MRI-PDFF, magnetic resonance imaging-proton density fat fraction.

The same 75 patients were separately stratified into S0, ≥ S1, and ≥ S2 grades according to two different MRI-PDFF threshold criteria: the Primary Set and the alternative set, as no consensus thresholds have been established by AASLD or EASL. In the primary set, thresholds derived from a recent large meta-analysis were used: S0 (< 5.5%), ≥S1 (≥5.5%), ≥S2 (≥15.5%), and S3 (≥20.5%) (19). In the Alternative Set, thresholds based on original histology-correlated derivation studies were applied: S0 (< 6.4%), ≥S1 (≥6.4%), and ≥S2 (≥17.4%) (20). The primary set was used for the main statistical analyses and diagnostic performance comparisons, while the alternative set served as a sensitivity analysis to assess the robustness of the comparative findings between UDFF and CT(L/S) to threshold selection (Figure 1).

### 2.2 Data collection

Patient characteristics including age, sex, etiology of liver disease, body mass index (BMI), skin-to-capsule distance (SCD), and laboratory parameters were collected. All participants underwent NCCT, ultrasound (UDFF and Automatic Point Shear Wave Elastography [Auto-pSWE]), and MRI-PDFF within 1 week. Imaging parameters included liver and spleen CT attenuation, MRI-PDFF, UDFF, and LSM values.

### 2.3 Ultrasound procedure

Ultrasound was conducted using Siemens ACUSON Sequoia system equipped with 5C1 (1.0–5.7 MHZ) convex abdominal probe and DAX deep abdominal probe (1.0–3.5 MHz). Patients fasted 4 h at least and were scanned supine with the right arm raised. Liver parenchyma, capsule smoothness, and SCD were assessed using the 5C1 probe. UDFF and Auto-pSWE were then underwent with the DAX probe, yielding simultaneous UDFF (%) and LSM (kPa) values (more detailed in Supplementary material, Part 2). For inter- and intra-operator repeatability, all examinations were independently performed by two sonographers: doctor 1 (>10 years' experience) and doctor 2 (2 years' experience). Doctor 1 repeated the measurement in all cases. All operators were blinded to clinical and imaging results.

### 2.4 MRI-PDFF procedure

MRI was performed on a 1.5-T scanner (Signa Voyager; GE HealthCare) using a torso coil. Fat fraction maps were generated using a multi-echo Dixon technique. Measurements were obtained from liver segments V and/or VIII, and the median value of three measurements was recorded for analysis (more detailed in Supplementary material, Part 2). All measurements were conducted by a radiologist with 10 years of experience, blinded to other findings.

### 2.5 NCCT procedure

Chest NCCT was performed using Siemens Somatom Definition AS (64-slice) or Philips Brilliance I scanners (128-slice) at end-inspiration at a low-radiation-dose and cover the entire liver and spleen. Dual-window reconstructions (soft-tissue and lung windows) were archived in PACS for evaluation (more detailed in Supplementary material, Part 2).

Hepatic and splenic attenuation were measured using mediastinal window settings (width: 350 HU; level: 50 HU) on PACS. In the liver, CT attenuation was measured by placing three 1.5-cm^2^ non-overlapping ROIs in segments S5 or/and S8 at the porta hepatis level, and the values were averaged to obtain the mean hepatic attenuation. In the spleen, three ROIs of the same size were placed in the upper, middle, and lower portions, and their CT attenuation values were averaged to obtain the mean spleen attenuation. CT(L/S) was calculated as: CT(L/S) = mean hepatic attenuation/mean splenic attenuation (10).

For assessment of inter- and intra-operator repeatability, two radiologists (doctor 3 with over 10 years' experience, and doctor 4 with 2 years' experience) independently measured all patients. Doctor 3 repeated the measurements in a separate session, with all measurements performed blinded to other findings.

### 2.6 Statistical analysis

Continuous variables are presented as median with interquartile range (IQR), categorical variables as counts and percentages. Group differences were evaluated with Kruskal–Wallis tests and *post-hoc* Mann–Whitney tests for continuous variables, and chi-square or Fisher's exact tests for categorical variables. Spearman's correlation coefficients assessed relationships among UDFF, CT(L/S), LSM, and MRI-PDFF. The minimum detectable area under the curve (AUC) under the achieved sample size was calculated using the Hanley-McNeil method (α = 0.05, power = 0.80, null hypothesis AUC = 0.5). Diagnostic performance was evaluated using Receiver operating characteristic (ROC) curves, and AUCs were compared using the bootstrap test (2,000 BCa resamples; random seed = 123). Agreement and reliability were evaluated by intraclass correlation coefficient (ICC). Bland–Altman analysis with linear regression of the differences on the means was performed to assess fixed and proportional bias. Grading consistency by Cohen's kappa. The Youden index was used to determine the optimal cutoff values for UDFF and CT(L/S) for detecting MRI-PDFF ≥ 5.5%. Based on these cutoffs, univariate and multivariate binary logistic regression were performed to identify determinants of HS as defined by UDFF and CT(L/S). Variables that were significant in univariate analysis (*p* < 0.05) were included in multivariate models. Odds ratios (ORs) and 95% confidence intervals (Cis) were estimated. Data were analyzed using SPSS 27.0.1 and RStudio 4.3.0, with statistical significance set at *p* < 0.05.

## 3 Results

### 3.1 Patient characteristics

Three patients were excluded (one splenectomy, two timing violations), yielding 75 patients (33 males, 42 females) for final analysis. Thirty-five (46.7%) were examined using the Siemens Somatom Definition AS (64-slice) scanner, and 40 (53.3%) using the Philips Brilliance I (128-slice) scanner. In the Primary Criteria Set Patients were divided into two groups based on MRI-PDFF ≥ 5.5%: non-HS (*n* = 18) and HS (*n* = 57). According to the etiology of liver disease (ELD), 27 had CHB (36.00%), of whom nine had concurrent MASLD, eight had ALD (10.67%), and 40 had MASLD (53.33%).

Compared to the HS group, non-HS group patients were significantly higher age and proportion of CHB, but lower BMI and SCD (all *p* < 0.05). Laboratory data showed that the non-HS group had lower triglycerides (TG) and hemoglobin A1c (HbA1c), and higher high-density lipoprotein cholesterol (HDL-C) levels (all *p* < 0.05). Imaging parameters revealed significantly higher UDFF and lower CT(L/S) in the HS group (both *p* < 0.05), but no differences in LSM (Supplementary material, Part 3: Supplementary Table S2).

**Figure 2:**
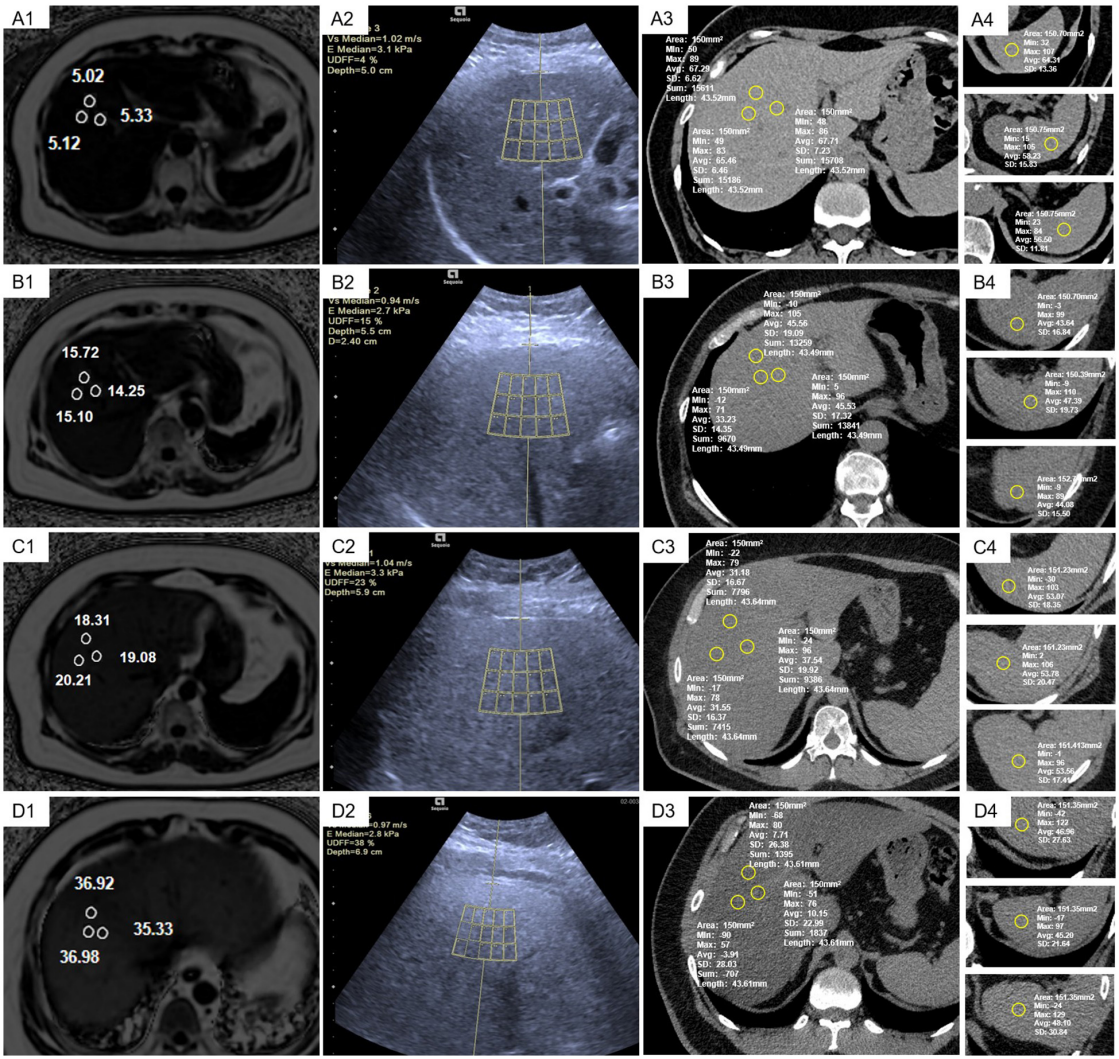
Representative images of MRI-PDFF, UDFF, CT(L/S) in four subjects. **(A1–A4)** A 61-year-old female patient presented with 5.12% MRI-PDFF, 4% UDFF, 1.1 CT(L/S), corresponding to S0; **(B1–B4)** a 67-year-old female patient presented with: 15.10% MRI-PDFF, 15% UDFF, 0.9 CT(L/S), representing S1; **(C1–C4)** a 61-year-old female patient presented with: 19.08% MRI-PDFF, 23% UDFF, 0.6 CT(L/S), representing S2; **(D1–D4)** a 37-year-old male patient presented with: 36.92% MRI-PDFF, 38% UDFF, 0.1 CT(L/S), representing S3. MRI-PDFF, magnetic resonance imaging-proton density fat fraction; UDFF, ultrasound-derived fat fraction; CT(L/S), CT liver-to-spleen attenuation ratio.

When patients were further classified into S0–S3 grades based on MRI-PDFF criteria. As HS grade increased, UDFF values increased, while CT(L/S) values decreased. Both UDFF and CT(L/S) demonstrated statistically significant differences across all grades (*p* < 0.001), but not for LSM (*p* = 0.145; Table 1). Representative images of MRI-PDFF, UDFF, and CT(L/S) for individuals with varying degrees of HS are shown in Figure 2.

**Table 1:** Comparison parameters UDFF, LSM, and CT (L/S) values in different grades of hepatic steatosis.

Parameters	S0	S1	S2	S3	χ^2^	*p-*value
*n*	18	30	11	16		
UDFF (%)	4.00 (3.25–5.75)	10.50 (9.00–13.00)	21.00 (14.50–23.00)	26.00 (22.25–35.00)	54.156	<0.001
LSM (kPa)	4.25 (3.18–5.90)	4.00 (3.32–5.38)	3.40 (3.10–4.30)	3.05 (2.80–3.82)	5.404	0.145
CT(L/S)	1.30 (1.12–1.50)	1.10 (0.90–1.40)	0.60 (0.55–0.75)	0.25 (0.18–0.35)	44.682	<0.001

UDFF, ultrasound-derived fat fraction; LSM, liver stiffness measurement; CT(L/S), CT liver-to-spleen attenuation ratio.

### 3.2 Correlation between UDFF, LSM, CT(L/S), and MRI-PDFF

**Figure 3:**
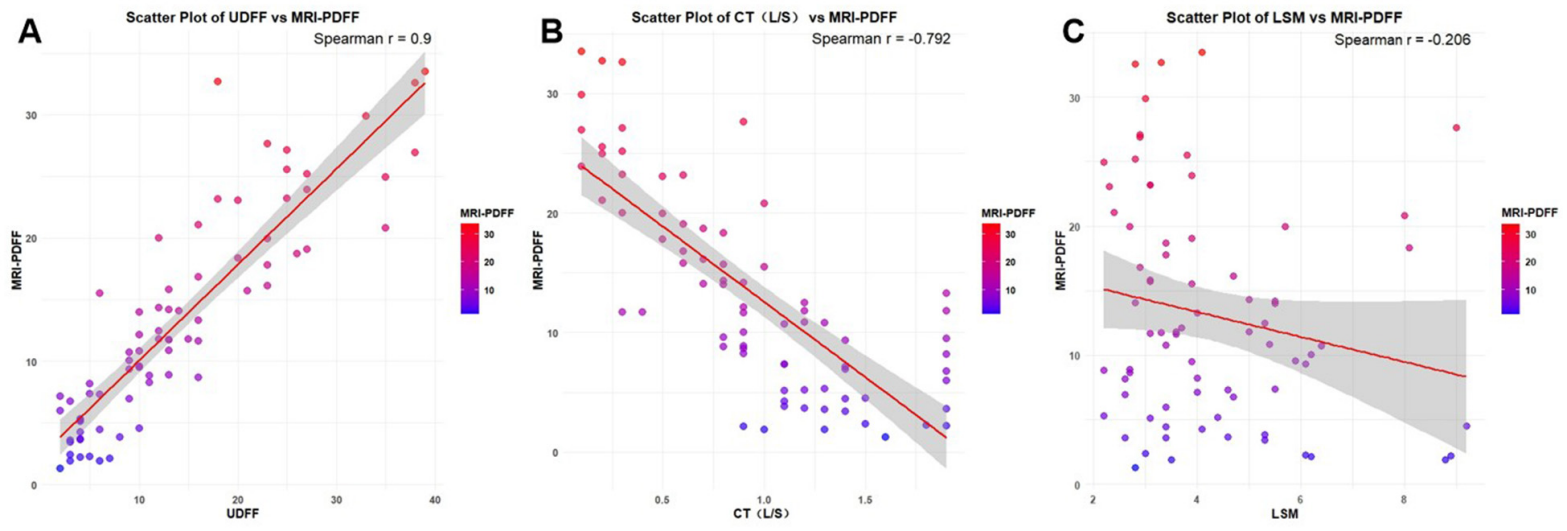
The correlations between UDFF, CT(L/S), and LSM with MRI-PDFF values. Red lines indicate lines of best fit with 95% confidence intervals. **(A)** UDFF showed a strong positive correlation with MRI-PDFF, with values increasing proportionally. **(B)** CT(L/S) exhibited a significant negative correlation with MRI-PDFF, with values decreasing as MRI-PDFF increased. **(C)** LSM showed no significant correlation with MRI-PDFF. MRI-PDFF, magnetic resonance imaging-proton density fat fraction; UDFF, ultrasound-derived fat fraction; LSM, liver stiffness measurement; CT(L/S), CT liver-to-spleen attenuation ratio.

Spearman's correlation coefficients analysis revealed a strong correlation between UDFF and MRI-PDFF (*r* = 0.900, 95% CI: 0.847–0.936, *p* < 0.001) and a significant inverse correlation between CT(L/S) and MRI-PDFF (*r* = −0.792, 95% CI: −0.863 to −0.689, *p* < 0.001). The correlation between LSM and MRI-PDFF was not significant (*r* = −0.206, 95% CI: −0.413 to 0.022, *p* = 0.076; Figure 3).

### 3.3 Diagnostic performance comparison between UDFF and CT(L/S)

Using the Hanley–McNeil method, the minimum detectable AUCs were 0.708 for HS ≥ S1 and 0.688 for HS ≥ S2 (α = 0.05, power = 0.80).

**Figure 4:**
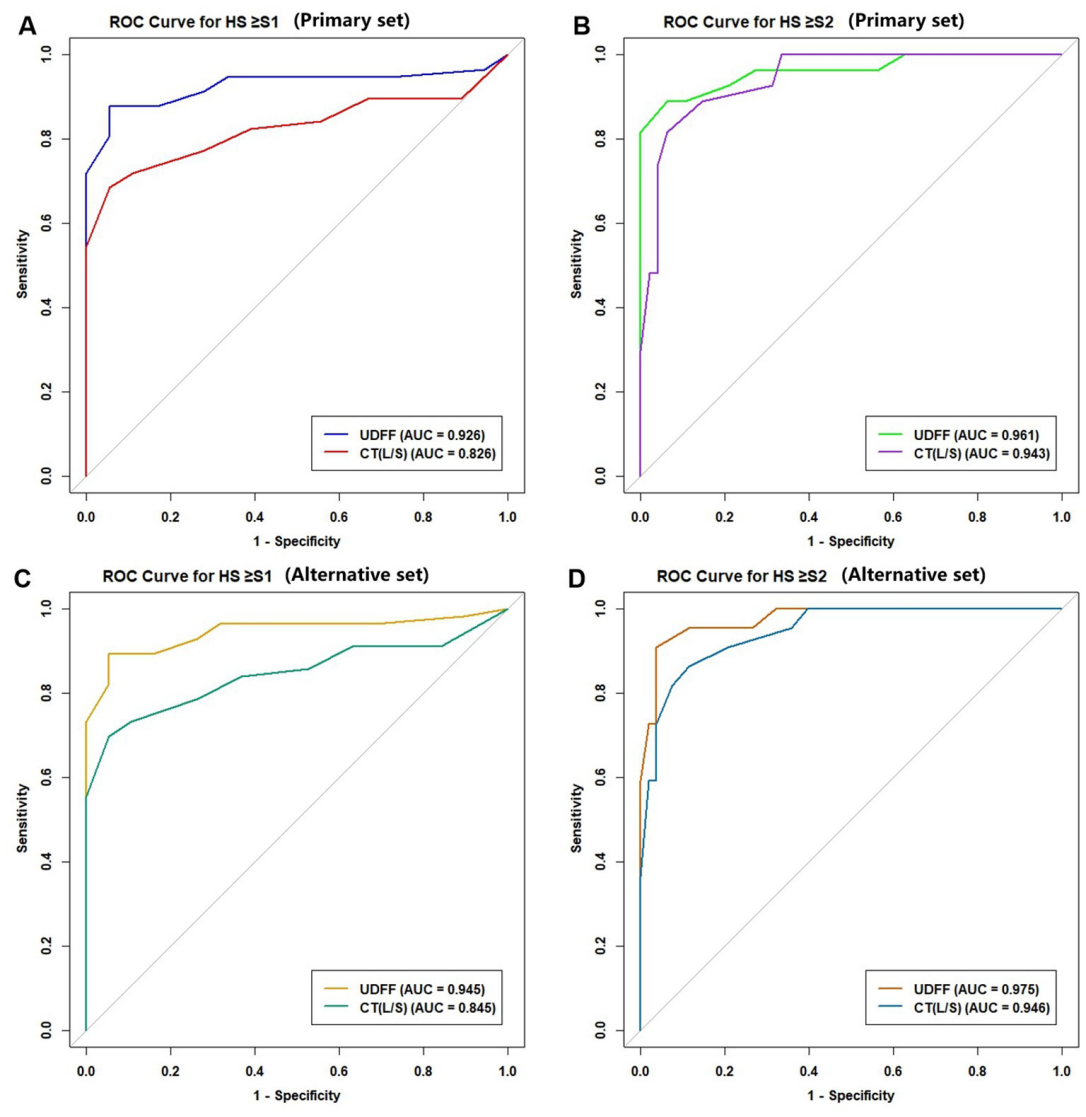
ROC curves in UDFF and CT(L/S) for detecting HS in primary set and alternative set, respectively. Primary set, **(A)** HS ≥ S1, UDFF and CT(L/S) demonstrated AUCs of 0.926 (blue) and 0.826 (red), respectively; **(B)** HS ≥ S2, UDFF and CT(L/S) showed AUCs of 0.961 (green) and 0.943 (purple), respectively. Alternative set, **(C)** HS ≥ S1, UDFF and CT(L/S) demonstrated AUCs of 0.945 (golden) and 0.845 (cyan), respectively; **(D)** HS ≥ S2, UDFF and CT(L/S) showed AUCs of 0.975 (brown) and 0.946 (dark blue), respectively. ROC, receiver operating characteristic; MRI-PDFF, magnetic resonance imaging-proton density fat fraction; UDFF, ultrasound-derived fat fraction; LSM, liver stiffness measurement; CT(L/S), CT liver-to-spleen attenuation ratio; HS, hepatic steatosis.

Primary set (MRI-PDFF thresholds: ≥5.5% for ≥S1; ≥15.5% for ≥S2). For HS ≥ S1, UDFF showed a higher AUC than CT(L/S) (0.926 [95% CI: 0.866–0.987] vs. 0.826 [95% CI: 0.735–0.916]; bootstrap *p* = 0.012). At a cutoff of 8.5%, UDFF achieved 87.72% sensitivity, 94.44% specificity, 98.04% positive predictive value (PPV), and 70.83% negative predictive value (NPV). For CT(L/S) (cutoff 0.95), these values were 68.42%, 94.44%, 97.50%, and 48.57%, respectively. For HS ≥ S2, UDFF and CT(L/S) had comparable AUCs (0.961 [95% CI: 0.913–1.000] vs. 0.943 [95% CI: 0.895–0.992]; bootstrap *p* = 0.506). At a cutoff of 15.5%, UDFF gave 88.89% sensitivity, 93.75% specificity, 88.89% PPV, and 93.75% NPV. For CT(L/S) (cutoff 0.75), these were 81.48%, 93.75%, 88.00%, and 90.00%, respectively (Table 2, Figures 4A, B).

**Table 2:** Diagnostic performance of UDFF and CT(L/S) for detecting hepatic steatosis in primary set and alternative set.

Criteria and parameter	Cut-off values	AUC (95%CI)	Sensitivity	Specificity	PPV	NPV
Primary set (MRI-PDFF ≥ 5.5%)						
UDFF	8.5%	0.926 (0.866–0.987)	87.72%	94.44%	98.04%	70.83%
CT(L/S)	0.95	0.826 (0.735–0.916)	68.42%	94.44%	97.50%	48.57%
*p-*value		0.012				
Alternative set (MRI-PDFF ≥ 6.4%)						
UDFF	8.5%	0.945 (0.895–0.995)	89.29%	94.74%	98.04%	75.00%
CT(L/S)	0.95	0.845 (0.760–0.931)	69.64%	94.74%	97.50%	51.43%
*p-*value		0.016				
Primary set (MRI-PDFF ≥ 15.5%)						
UDFF	15.5%	0.961 (0.913–1.000)	88.89%	93.75%	88.89%	93.75%
CT(L/S)	0.75	0.943 (0.895–0.992)	81.48%	93.75%	88.00%	90.00%
*p-*value		0.506				
Alternative set (MRI-PDFF ≥ 17.4%)						
UDFF	17%	0.975 (0.944–1.000)	90.91%	96.23%	90.91%	96.23%
CT(L/S)	0.75	0.946 (0.896–0.995)	86.36%	88.68%	76.00%	94.00%
*p-*value		0.323				

AUC, area under the curve; CI, confidence interval; CT(L/S), CT liver-to-spleen attenuation ratio; MRI-PDFF, magnetic resonance imaging-proton density fat fraction; NPV, negative predictive value; PPV, positive predictive value; UDFF, ultrasound-derived fat fraction.

Alternative set (MRI-PDFF thresholds: ≥6.4% for ≥S1; ≥17.4% for ≥S2). For HS ≥ S1, UDFF again outperformed CT(L/S) (AUC: 0.945 [95% CI: 0.895–0.995] vs. 0.845 [95% CI: 0.760–0.931]; bootstrap *p* = 0.016). At the same 8.5% cutoff, UDFF yielded 89.29% sensitivity, 94.74% specificity, 98.04% PPV, and 75.00% NPV. For CT(L/S) (cutoff 0.95), values were 69.64%, 94.74%, 97.50%, and 51.43%. For HS ≥ S2, no significant difference was observed (UDFF AUC: 0.975 [95%CI: 0.944–1.000]; CT(L/S): 0.946 [95%CI: 0.896–0.995]; bootstrap *p* = 0.323). Using a 17% cutoff, UDFF gave 90.91% sensitivity, 96.23% specificity, 90.91% PPV, and 96.23% NPV. For CT(L/S) (cutoff 0.75), these were 86.36%, 88.68%, 76.00%, and 94.00%, respectively (Table 2, Figures 4C, D).

### 3.4 Agreement analysis

**Figure 5:**
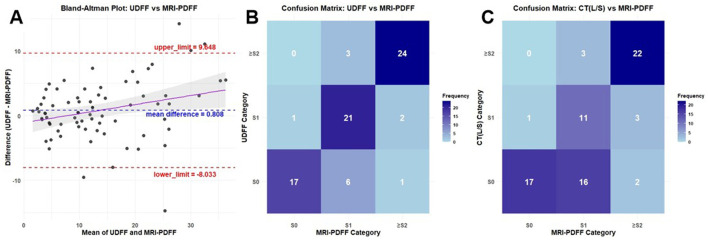
Agreement analysis. **(A)** Bland–Altman difference plot shows comparison of overall UDFF and MRI-PDFF, with a mean bias of 0.808% (blue dashed line) and 95% limits of agreement ranging from −8.03 to 9.65 (red dashed lines), linear regression model of association between differences and means shows proportional bias (*p* = 0.016), with greater overestimation of MRI PDFF by UDFF with increasing fat content (purple line shows linear line of best fit for regression model.). **(B,C)** Consistency heatmaps demonstrate superior diagnostic consistency of UDFF over CT(L/S) for grading diagnosis. (Kappa interpretation ≤0.20: poor; 0.21–0.40: fair; 0.41–0.60: moderate; 0.61–0.80: good; 0.81–1.00: very good.) MRI-PDFF, magnetic resonance imaging-proton density fat fraction; UDFF, ultrasound-derived fat fraction; CT liver-to-spleen attenuation ratio.

UDFF demonstrated good agreement with MRI-PDFF in quantitative analysis (ICC = 0.878, 95% CI: 0.813–0.921, *p* < 0.05). Bland-Altman analysis revealed a mean bias of 0.808% between UDFF and MRI-PDFF values. Linear regression analysis of differences and means showed a significant proportional bias (regression coefficient = 0.140, 95% CI: 0.057–2.463; *p* = 0.016), with the difference between the two methods increasing at higher fat fraction values. For grading diagnosis, Kappa analysis revealed good agreement between UDFF and MRI-PDFF (κ = 0.740, 95% CI: 0.600–0.876, *p* < 0.05), while CT(L/S) showed only moderate agreement (κ = 0.516, 95% CI: 0.363–0.661, *p* < 0.05; Figure 5).

### 3.5 Reliability analysis of UDFF, CT(L/S) inter- and intra-operators

UDFF demonstrated excellent inter-operator (ICC = 0.949, 95% CI: 0.910–0.970, *p* < 0.05) and intra-operator (ICC = 0.967, 95% CI: 0.948–0.979, *p* < 0.05) reliability. CT(L/S) showed lower inter-operator (ICC = 0.841, 95% CI: 0.759–0.897, *p* < 0.05) and intra-operator (ICC = 0.886, 95% CI: 0.826–0.927, *p* < 0.05) consistency (Supplementary material, Part 3: Supplementary Figure S1).

### 3.6 Correlations analysis of UDFF and CT(L/S) with clinical parameters

Spearman's (for continuous variables) or Pearson's (for categorical variables) correlation coefficients were used to assess relationships among UDFF, CT(L/S), and clinical parameters. All reported as the correlation coefficient *r* (Supplementary material, Part 3: Supplementary Table S3, Supplementary Figure S2).

UDFF showed significant negative correlation with age (*r* = −0.528, 95%CI: −0.678 to −0.335, *p* < 0.001), HDL-C (*r* = −0.290, 95%CI: −0.481 to −0.061, *p* = 0.011), and CVH (*r* = −0.515, 95%CI: −0.664 to −0.326, *p* < 0.001). Significant positive correlations were with BMI (*r* = 0.787, 95% CI: 0.678–0.862, *p* < 0.001), SCD (*r* = 0.756, 95% CI: 0.635–0.841, *p* < 0.001), TG (*r* = 0.513, 95%CI: 0.318–0.667, *p* < 0.001), HbA1c (*r* = 0.313, 95%CI: 0.086–0.509, *p* = 0.006), γ-GGT (*r* = 0.308, 95% CI: 0.081–0.505, *p* = 0.007), ALT (*r* = 0.278, 95% CI: 0.048–0.481, *p* = 0.016), and MASLD (*r* = 0.409, 95% CI: 0.200–0.582, *p* < 0.001). No significant correlations were detected between UDFF and other parameters (all *p* > 0.05).

CT(L/S) demonstrated negative correlation with MASLD (*r* = −0.469, 95% CI: −0.629 to −0.271, *p* < 0.001), BMI (*r* = −0.680, 95% CI: −0.788 to −0.531, *p* < 0.001), SCD (*r* = −0.594, 95% CI: −727 to −0.418, *p* < 0.001), TG (*r* = −0.411, 95% CI: −0.588 to −0.196, *p* < 0.001), ALT (*r* = −0.388, 95% CI: −0.570 to −0.170, *p* < 0.001), AST (*r* = −0.265, 95% CI: −0.469 to −0.034, *p* = 0.022), γ-GGT (*r* = −0.255, 95% CI: −0.461 to −0.023, *p* = 0.027), HbA1c (*r* = −0.233, 95% CI: −0.442 to 0.000, *p* = 0.044). Positive correlations were identified for CVH (*r* = 0.490, 95% CI: 0.296–0.645, *p* < 0.001), Age (*r* = 0.472, 95% CI: 0.268–0.636, *p* < 0.001), LSM (*r* = 0.275, 95% CI: 0.044–0.477, *p* = 0.017), and HDL-C (*r* = 0.235, 95% CI: 0.002–0.444, *p* = 0.042). No significant correlations were detected between CT(L/S) and other parameters (all *p* > 0.05).

### 3.7 Determinants of HS as defined by UDFF and CT(L/S)

The optimal cutoff values for detecting MRI-PDFF ≥ 5.5% were 8.5% for UDFF and 0.95 for CT(L/S). Multicollinearity diagnostics showed that all variance inflation factors (VIF) values were below 5 (UDFF: 1.050–3.955; CT(L/S): 1.105–4.010), indicating no significant collinearity (Supplementary material, Part 3, Supplementary Table S4). Univariate binary logistic regression analyses were performed on the factors significantly associated with UDFF and CT(L/S). All variables with *p* < 0.05 in univariate analysis or those considered clinically important CT(L/S) including CT scanner type were entered into the multivariate logistic regression model. The findings revealed that TG level was the determinant factor for UDFF (OR = 8.510, 95% CI: 1.318–54.956, *p* < 0.05), and there is no determinant factor for CT(L/S) (Table 3). To assess the robustness of the association between TG and UDFF, we *p*erformed bootstrap resampling with 1,000 iterations. The bootstrap analysis confirmed that TG remained a significant independent determinant of UDFF (bootstrap OR = 8.510, 95% BCa CI: 2.16–38.42).

**Table 3:** Determinants of hepatic steatosis as defined by UDFF and CT(L/S).

Factor	UDFF				CT(L/S)			
	Univariate analysis		Multivariate analysis		Univariate analysis		Multivariate analysis	
	OR (95% CI)	*p-*Value	OR (95% CI)	*p-*Value	OR (95% CI)	*p-*Value	OR (95% CI)	*p-*Value
BMI	1.641 (1.297, 2.077)	< 0.001	1.251 (0.910, 1.172)	0.168	1.245 (1.120, 1.384)	< 0.001	1.161 (0.965, 1.398)	0.114
SCD	1.374 (1.182, 1.596)	< 0.001	1.089 (0.828,1.433)	0.540	1.162 (1.073, 1.260)	< 0.001	1.021 (0.900, 1.159)	0.744
CHB	14.000 (4.339, 45.167)	< 0.001	4.753 (0.031,732.289)	0.544	11.846 (3.710, 37.830)	< 0.001	0.650 (0.054, 7.859)	0.734
MASLD	0.120 (0.038, 0.380)	< 0.001	0.575 (0.004,79.231)	0.826	0.100 (0.035, 0.290)	< 0.001	0.082 (0.007, 0.995)	0.050
Age	0.920 (0.879, 0.963)	< 0.001	1.002 (0.915,1.097)	0.969	0.933 (0.898, 0.970)	< 0.001	0.976 (0.918, 1.037)	0.427
TG	6.753 (2.458, 18.550)	< 0.001	8.510 (1.318,54.956)	0.024	1.557 (1.051–2.305)	0.027	1.121 (0.576, 2.185)	0.736
HDL-C	0.271 (0.088, 0.833)	0.023	0.333 (0.035,3.204)	0.341	0.260 (0.080, 0.843)	0.025	0.330 (0.058, 1.885)	0.212
HbA1c	1.300 (1.032, 1.638)	0.026	1.176 (0.780,1.774)	0.439	1.215 (1.006, 1.467)	0.043	1.206 (0.906, 1.605)	0.200
ALT	0.997 (0.990, 1.005)	0.497	0.999 (0.981,1.017)	0.907	0.999 (0.992, 1.007)	0.863	1.010 (0.989, 1.031)	0.352
γ-GGT	1.007 (0.996, 1.017)	0.212	0.994 (0.980,1.009)	0.427	1.000 (0.995, 1.005)	0.994	1.001 (0.991, 1.011)	0.863
LSM					0.756 (0.567, 1.009)	0.057	1.179 (0.736, 1.889)	0.494
AST					1.000 (0.987, 1.014)	0.986	0.979 (0.942, 1.017)	0.269
CT scanner					0.944 (0.381, 2.340)	0.902	0.408 (0.101, 1.647)	0.208

UDFF, ultrasound-derived fat fraction; CT(L/S), CT liver-to-spleen attenuation ratio; CI, confidence interval; OR, odds ratio; BMI, body mass index; SCD, skin-to-capsulate distance; CHB, chronic viral hepatitis B; MASLD, metabolic dysfunction-associated steatotic liver disease; TG, triglyceride; HDL-C, high-density lipoprotein cholesterol; HbA1c, haemoglobinA1c; ALT, alanine aminotransferase; γ-GGT, γ-glutamyl transpeptidase; LSM, liver stiffness measurement; AST, aspartate aminotransferase.

OR > 1 indicates higher odds of being classified as having steatosis by UDFF (≥8.5%) or CT(L/S) ( ≤ 0.95).

## 4 Discussion

NCCT offers a reliable, non-invasive, and widely accessible alternative to MRI-PDFF for assessing HS (21, 22). CT(L/S) demonstrates strong correlation with histopathological severity and is less influenced by technical variability (12). Meanwhile, the emerging QUS technique UDFF also shows a significant correlation with MRI-PDFF (*r* = 0.798–0.876) (23, 24). Although both CT(L/S) and UDFF show promise as non-invasive quantitative tools, direct comparative studies are scarce, warranting further investigation into their relative clinical utility and diagnostic performance.

In this study, we observed that as the grade of HS increased, CT(L/S) values progressively decreased, demonstrating a significant negative correlation with MRI-PDFF (*r* = −0.792), while UDFF values correspondingly increased, showing a strong positive correlation with MRI-PDFF (*r* = 0.900). For detecting HS ≥ S1 and ≥S2, the optimal CT(L/S) cut-offs were 0.95 (AUC = 0.826) and 0.75 (AUC = 0.943), respectively. These cut-off values are comparable to those reported by Tang et al. (22) (≥S1: 1.0, ≥S2: 0.7), and the AUCs were superior to the results from Byun et al. (12), who used liver biopsy as the reference standard (AUCs of 0.732 and 0.925 for HS ≥ 5% and ≥33%, respectively). Similarly, our UDFF cut-offs for HS ≥ S1 (8.5%, AUC = 0.926) and ≥S2 (15.5%, AUC = 0.961) were consistent with the values reported by Huang et al. (7.6% and 15.9%, AUCs = 0.90) (18), while demonstrating improved diagnostic accuracy. This study builds on the evolution from conventional ultrasound toward QUS techniques such as controlled attenuation parameter (CAP), attenuation imaging (ATI), and UDFF. CAP and ATI are single-parameter techniques that estimate only the attenuation coefficient, assuming constant backscatter, in contrast, UDFF is a dual-parameter technique that simultaneously estimates both attenuation and backscatter coefficients. By accounting for backscatter variations, which become non-negligible at higher fat fractions, UDFF theoretically offers more accurate and robust fat fraction estimation across a wider range of steatosis grades (14).

While both ultrasound and CT are routinely available in clinical practice and demonstrate good diagnostic performance for HS screening. However, direct comparisons between QUS and CT remain limited. For instance, one study comparing ATI (a QUS technique) with CT liver-spleen attenuation difference, showed that ATI (AUC = 0.914) outperformed CT (AUC = 0.807) for detecting HS ≥ 5%, while both performed comparably for HS ≥ 33% (AUCs: 0.914 vs. 0.887) (25).

To our knowledge, no study has directly compared the diagnostic performance of UDFF and CT(L/S) for detecting or grading hepatic steatosis. The key findings as follow: for detecting HS ≥ S1, UDFF demonstrated significantly superior diagnostic efficacy compared to CT(L/S), with AUCs of 0.926 and 0.826, respectively (*p* < 0.05), and higher sensitivity (87.72% vs. 68.42%). For HS ≥ S2, the diagnostic performance of the two methods was comparable, with AUCs of 0.961 for UDFF and 0.943 for CT(L/S) (*p* > 0.05). Notably, although UDFF achieved an AUC of 0.961 for detecting HS ≥ S2, the relatively wide 95% CI (0.913–1.000) reflects the uncertainty inherent to the current sample size. Nevertheless, the lower bound of the CI (0.913) remains substantially higher than the minimum detectable AUC calculated for this sample size (0.688), supporting the robustness of the observed diagnostic accuracy. Beyond AUC, UDFF showed good quantitative agreement with MRI-PDFF (ICC = 0.878). Bland-Altman analysis revealed a small fixed bias (mean difference = 0.808%) but significant proportional bias (regression coefficient = 0.140, *p* = 0.016), indicating that the difference between UDFF and MRI-PDFF increases at higher fat fraction values. This finding is consistent with the observations of Dillman JR (15). The proportional bias may reflect both statistical instability due to the limited sample size in the ≥S2 subgroup (*n* = 27). Consequently, UDFF cannot fully substitute for MRI-PDFF in quantitative assessment of HS, particularly at high fat fractions. UDFF outperformed CT(L/S) in grading diagnostic agreement (κ = 0.740 vs. κ = 0.517), thus, proportional bias does not affect the diagnostic performance of UDFF for detecting steatosis grades (AUC ≥ 0.926). In summary, UDFF demonstrates excellent performance for HS grading, but its accuracy for quantitative fat fraction analysis requires further validation, particularly in patients with high-grade HS. Furthermore, UDFF exhibited excellent inter-operator (ICC = 0.949) and intra-operator (ICC = 0.967) reproducibility, exceeding the corresponding values for CT(L/S) (ICCs = 0.841 and 0.886), the latter being consistent with previously reported test-retest reliability for CT (ICC range: 0.553–0.913) (26).

Analysis of clinical characteristics showed that the HS group had a significantly lower median age than the non-HS group, suggesting a trend toward younger onset of HS (1). The non-HS group had a higher prevalence of CHB, which may be attributed to the metabolic protective effects and inhibition of hepatic lipogenesis associated with hepatitis B virus infection (4, 27). Additionally, the HS group exhibited significantly higher levels of BMI, SCD, TG, and HbA1c, along with lower HDL-C levels, further confirming the close association between obesity, glucose and lipid metabolism disorders (1, 2). Regression analysis identified TG level (OR = 8.510, 95% CI: 1.318–54.956, *p* < 0.05) as an independent determinant of UDFF, whereas no independent determinant—including both patient-related factors and scanner type—was identified for CT(L/S). Multicollinearity diagnostics using variance inflation factors (VIF) showed that all VIF values were below 5 [UDFF: 1.050–3.955; CT(L/S): 1.105–4.010], indicating no significant collinearity among the independent variables. These findings confirm that the regression estimates are stable and reliable. Although the wide confidence interval raised concerns about estimate stability, bootstrap sensitivity analysis yielded a substantially narrower 95% CI (2.16–38.42) that remained far above unity, confirming TG level as a robust independent determinant of UDFF. These findings differ from Flagiello et al. (28), who reported age, hypertension, and HbA1c—in addition to TG—as independent determinants of UDFF. The discrepancy likely attributable to differences in study population, methodology, and sample size.

Whether HS affects liver stiffness remains controversial, and few studies have utilized LSM for quantitative assessment of steatosis (29, 30). Although LSM showed no significant differences between the non-HS and HS groups or across steatosis grades, and was not significantly correlated with MRI-PDFF (*r* = −0.206, *p* = 0.076), this negative finding does not exclude a potential independent effect of fat accumulation on liver stiffness. The relationship between LSM and steatosis may be confounded by coexisting liver fibrosis, which is the dominant determinant of LSM (31). In the presence of such confounding, a positive association would have required further stratification by fibrosis stage (e.g., non-significant vs. significant fibrosis) to isolate the independent contribution of steatosis. However, due to the limited sample size, such stratified analysis was not feasible. Given that LSM assessment was exploratory in nature, we elected not to perform underpowered subgroup analyses that could yield unreliable estimates.

A key strength of this study is its direct comparison of the diagnostic performance of the novel UDFF technique with the established CT-based method. To our knowledge, this represents the first head-to-head evaluation, offering new insights for HS assessment in CLD.

The study has several limitations. First, our UDFF results are platform-dependent (Siemens ACUSON Sequoia), and therefore cannot be generalized to other QUS techniques (e.g., GE UGAP, Canon ATI, Samsung TAI) without direct cross-platform validation. Second, while our design intentionally avoided additional radiation exposure and reflected real-world clinical practice where NCCT examinations are already indicated, it may have introduced potential selection bias. Third, our findings are most directly applicable to opportunistic screening scenarios; validation in general chronic liver disease (CLD) populations without NCCT indications is warranted. Fourth, the cohort had a limited sample size, particularly for moderate-to-severe steatosis (≥S2) cases, and was from a single center in China, which may limit the generalizability of the findings.

## 5 Conclusions

In conclusion, as opportunistic screening tools using MRI-PDFF as the reference standard, UDFF showed superior performance for detecting HS ≥ S1 and better grading agreement/reliability, while diagnostic performance was comparable to CT(L/S) for HS ≥ S2. However, UDFF is restricted to the Siemens DAX probe, whereas CT(L/S) is equipment-independent at the cost of lower reliability. UDFF also shows good quantitative agreement with MRI-PDFF, though its consistency in high-grade steatosis requires further validation with larger sample sizes.

## Data Availability

The raw data supporting the conclusions of this article will be made available by the authors, without undue reservation.
