# Supplementary material for: Comparing ultrasound-derived fat fraction and CT for diagnosing hepatic steatosis: an MRI-PDFF reference study
Source: Front Med (Lausanne). 2026 Jun 10;13:1799359. doi: 10.3389/fmed.2026.1799359 (PMC13290692; doi:10.3389/fmed.2026.1799359)
Supplement: Supplementary file 1 [file Table_1.docx]

**Comparing Ultrasound-derived Fat Fraction and CT for Diagnosing Hepatic Steatosis: An MRI-PDFF Reference Study**

**Supplementary Material**

**Index**

| Part/Item | Description |
| --- | --- |
| Part 1 | Diagnostic criteria for common chronic liver disease |
| Part 2 | Methods |
| Part 3 | Supplementary tables and figures |
| Table S1 | Indicators of metabolic abnormality |
| Table S2 | characteristics of patient |
| Table S3 | Spearman's (for continuous variables) or Pearson's (for categorical variables) correlation coefficients between UDFF, CT(L/S) values and clinical parameters |
| Table S4 | Multicollinearity Diagnostics for UDFF and CT(L/S) Multivariate Linear Regression Model |
| FIGURE S1 | Reliability Analysis. |
| FIGURE S2 | Bar charts showing Spearman's (for continuous variables) or Pearson's (for categorical variables) correlation coefficients between UDFF (A) and CT(L/S) (B) and clinical parameters. |

**Part 1 Diagnostic criteria for common chronic liver disease**

**1、MASLD :** The adult criteria according to the multi-society Delphi consensus.

（1）Hepatic steatosis was confirmed through imaging studies or liver biopsy;

（2）Incorporate at least one of the following indicators of metabolic abnormality (Table S1)：

**Table S1** Indicators of metabolic abnormality

| Metabolic risk factor | Adult Criteria |
| --- | --- |
| Overweight or Obesity | Body mass index ≥ 25 kg/m²  **or** waist circumference > 94 cm (M) 80 cm (F)  **or** ethnicity adjusted equivalent; |
| Dysglycaemia or type 2 diabetes | Fasting serum glucose ≥ 5.6 mmol/L (100 mg/dl)  **or** 2-hour post-load glucose levels ≥ 7.8 mmol/L (≥ 140 mg/dl)  **or** HbA1c ≥ 5.7% (39mmol/L)  **or** type 2 diabetes  **or** treatment for type 2 diabetes |
| Plasma triglycerides | ≥ 1.70 mmol/L (150 mg/dl)  **or** lipid lowering treatment |
| HDL-cholesterol | ≤ 1.0 mmol/L (40 mg/dl) (M) and ≤ 1.3 mmol/L (50 mg/dl) (F)  **or** lipid-lowering treatment |
| Blood pressure | ≥ 130/85 mmHg  **or** specific antihypertensive drug treatment |

（3）Exclude other potential causes of hepatic steatosis, including excessive alcohol consumption, viral hepatitis, drug-induced liver injury, and inherited metabolic liver disorders.

**2、Chronic viral hepatitis B:** a persistent inflammatory condition of the liver resulting from long-term infection (lasting ≥ 6 months) with hepatitis B virus.

**3、Alcoholic liver disease：**Hepatic steatosis was confirmed through imaging studies or liver biopsy，and Excessive alcohol (consumption is defined as ethanol intake exceeding 420 g/week for men and 350 g/week for women.)

The amount of ethanol (in grams) can be calculated using the following formula: ethanol content (%) × volume of alcoholic beverage consumed (mL) × specific gravity of ethanol (0.8).

**Part 2 Methods**

**NCCT Procedure**

Participants with indications (such as respiratory symptoms, cancer screening, and health check-ups) for NCCT (chest/ abdomen) scans covering the entire liver and spleen were included. NCCT using standardized protocols (Tube voltage 120kV, tube current 100-216mAs, pitch 1, rotation time 0.5s, layer thickness 1.5mm, and layer spacing 1.5mm).

**MRI-PDFF Procedure**

All participants underwent MRI using standardized protocols after fasting for at least 4 hours, in the supine, head-first position. Hepatic proton density fat fraction (PDFF) maps were generated using a 1.5-T scanner with the iterative decomposition of water and fat with echo asymmetry and least-squares estimation quantitation (IDEAL-IQ) sequence, a multi-echo Dixon technique. The scan was performed during a single 20-second breath-hold with the following parameters: repetition time/echo time (TR/TE) = 11ms/5ms, and axial acquisition covering the entire liver. Additional parameters included a 5° flip angle, 8 mm slice thickness. PDFF maps were stored and analyzed. Three 1.0-cm^2^ non-overlapping ROIs were placed in liver segments V or/and VIII, while also avoiding blood vessels, biliary structures, and focal lesions. The mean MRI-PDFF value from each ROI was automatically calculated, and the median of three measurements was used for analysis.

**Ultrasound Procedure**

Participants with indications (such as epigastric discomfort, cancer screening, and health check-ups) for abdominal ultrasound. Ultrasound examinations were performed using a Siemens ACUSON Sequoia system (software version：Sequoia VA30C) with a DAX transducer (1.0-3.5MHz) under consistent preset parameters (mechanical index, 1.38; frame rate, 18 fps; transmit power, 95%; frequency, H Mid; dynamic range, 60 dB; tissue harmonic imaging enabled; speed of sound, 1540 m/s; Depth:＜15 cm. The DAX transducer is designed for both UDFF and Auto-pSWE techniques, enabling simultaneous assessment of hepatic fat content and liver stiffness measurements (LSM) in a single operation.

The UDFF technique simultaneously analyzes two acoustic parameters—ultrasound attenuation coefficient and backscatter coefficient—within a fixed 3×3 cm region of interest (ROI). Through a dedicated algorithm, it yields a UDFF value (%), providing an acoustically based estimation of hepatic fat content, like MRI-PDFF. Concurrently, the Auto‑pSWE technique acquires 15 LSM within the same ROI, which is divided into 15 sub‑ROIs. A reliable measurement requires at least ten valid sub‑ROI values. The median of the 10-15 LSM values (kPa) can be automatically calculated.

During examination, ensure that the B-mode ultrasound image is clearly visible. The ROI should be placed within the parenchyma of segment V or VIII, 1.5 cm from the liver capsule. Measurements were obtained during quiet suspended respiration, avoiding Valsalva maneuver, and ensuring that the ROI did not include large vessels, focal lesions. The entire procedure was repeated five times, and the median value of these measurements was used for final analysis.

**Part 3 supplementary tables and figures**

**Table S2** characteristics of patient.

| Characteristics | All | HS Group | non-HS Group | *p-*value |
| --- | --- | --- | --- | --- |
| n | 75 | 57 | 18 |  |
| Age, years | 49.00(34.50-58.50) | 41.00(31.00-55.00) | 57.50(51.50-65.75) | < 0.001 |
| Male, n (%) | 33(44.00%) | 24(42.10%) | 9(50.00%) | 0.595 |
| CHB, n (%) | 27 | 9(15.79%) | 18(100.00%) | < 0.001 |
| BMI, kg/m^2^ | 27.50(22.07-32.95) | 30.10(26.00-35.40) | 21.23(20.25-21.78) | < 0.001 |
| SCD, mm | 29.00(24.50-35.00) | 32.00(27.00-37.00) | 19.00(17.00-21.00) | < 0.001 |
| **Laboratory results** |  |  |  |  |
| WBC, 10^9^/L | 6.40(5.74-8.09) | 6.39(5.64-7.69) | 6.42(5.94-9.40) | 0.201 |
| PLT, 10^9^/L | 208.0(164.0-270.0) | 228.0(165.0-280.0) | 176.0(160.5-243.0) | 0.111 |
| AST, U/L | 28.00(21.00-35.35) | 28.00(21.00-36.00) | 29.50(23.00-34.50) | 0.891 |
| ALT, U/L | 33.00(19.50-57.50) | 33.00(21.00-58.00) | 30.50(16.00-37.50) | 0.318 |
| ALP, U/L | 92.00(74.00-113.50) | 88.0(74.0-108.0) | 107.5(75.0-132.8) | 0.193 |
| γ-GGT, U/L | 42.00(27.50-71.00) | 43.00(34.00-78.00) | 29.50(20.25-47.00) | 0.043 |
| TB, mmol/L | 12.60(9.85-15.40) | 12.60(10.00-15.50) | 11.90(8.47-15.20) | 0.682 |
| TG, mmol/L | 2.02(1.27-3.16) | 2.33(1.61-3.61) | 1.23(1.09-1.79) | < 0.001 |
| TC, mmol/L | 4.96(4.32-5.59) | 5.03(4.33-5.54) | 4.67(4.27-6.12) | 0.814 |
| HDL-C, mmol/L | 1.28(1.08-1.64) | 1.21(1.07-1.50) | 1.53(1.29-1.84) | 0.014 |
| LHL-C, mmol/L | 2.91(2.49-3.40) | 2.94(2.54-3.34) | 2.62(2.22-3.65) | 0.689 |
| HbA1c, % | 6.30(5.50-9.30) | 7.20(5.60-10.50) | 5.30(4.82-5.85) | < 0.001 |
| **Imaging parameters** |  |  |  |  |
| CT(L/S) | 0.90(0.60-1.30) | 0.80(0.40-1.10) | 1.30(1.13-1.50) | < 0.001 |
| UDFF, % | 12.00(6.00-20.00) | 14.0(10.0-23.0) | 4.00 (3.25-5.75) | < 0.001 |
| LSM, kPa | 3.70(3.00-5.30) | 3.60(2.90-5.00) | 4.25(3.17-5.90) | 0.250 |
| MRI-PDFF, % | 11.71(6.37-18.89) | 14.18(10.05-21.06) | 3.60(2.22-4.39) | < 0.001 |

Abbreviations: CHB, Chronic viral hepatitis B; BMI, body mass index; SCD, skin-to-capsulate distance; WBC, white blood cell; PLT, platelet count; AST, aspartate aminotransferase; ALT, alanine aminotransferase; ALP, alkaline phosphatase; γ-GGT, γ-glutamyl transpeptidase; TB, total bilirubin; TG, triglycerides; TC, total cholesterol; HDL-C, high-density lipoprotein cholesterol; LDL-C, low-density lipoprotein cholesterol; HbA1c, haemoglobinA1c; CT(L/S), CT liver-to-spleen attenuation ratio; UDFF, ultrasound-derived fat fraction; LSM, liver stiffness measurement; MRI-PDFF, magnetic resonance imaging-proton density fat fraction.

**Table S3** Spearman's (for continuous variables) or Pearson's (for categorical variables) correlation coefficients between UDFF, CT (L/S) values and clinical parameters

| Parameters | UDFF values | | CT (L/S) values | |
| --- | --- | --- | --- | --- |
|  | *r* (95% *CI*) | *p* values | *r* (95% *CI*) | *p* values |
| *Gender, Male | -0.064 (-0.287 to 0.165) | 0.585 | 0.044 (-0.185 to 0.268) | 0.709 |
| *CHB | -0.515 (-0.664 to -0.326) | ＜0.001 | 0.490 (0.296-0.645) | ＜0.001 |
| *ALD | 0.140 (-0.090 to 0.356) | 0.231 | -0.004 (-0.231 to 0.223) | 0.973 |
| *MASLD | 0.409 (0.200-0.582) | ＜0.001 | -0.469 (-0.629 to -0.271) | ＜0.001 |
| *CT scanner |  |  | 0.944 (0.381-2.340) | 0.902 |
| ^†^Age, years | -0.528 (-0.678 to -0.335) | ＜0.001 | 0.472 (0.268-0.636) | ＜0.001 |
| ^†^BMI, kg/m^2^ | 0.787 (0.678-0.862) | ＜0.001 | -0.680 (-0.788 to -0.531) | ＜0.001 |
| ^†^SCD, mm | 0.756 (0.635-0.841) | ＜0.001 | -0.594 (-0.727 to -0.418) | ＜0.001 |
| ^†^WBC, 10^9^/L | 0.010 (-0.224 to 0.243) | 0.932 | -0.099 (-0.325 to 0.137) | 0.397 |
| ^†^PLT, 10^9^/L | 0.173 (-0.063 to 0.391) | 0.137 | -0.182 (-0.399 to 0.053) | 0.117 |
| ^†^AST, U/L | 0.152 (-0.083 to 0.373) | 0.190 | -0.265 (-0.469 to -0.034) | 0.022 |
| ^†^ALT, U/L | 0.278 (0.048-0.481) | 0.016 | -0.388 (-0.570 to -0.170) | ＜0.001 |
| ^†^ALP, U/L | -0.088 (-0.315 to 0.149) | 0.453 | 0.051 (-0.185 to 0.281) | 0.665 |
| ^†^γ-GGT, U/L | 0.308 (0.081-0.505) | 0.007 | -0.255 (-0.461 to -0.023) | 0.027 |
| ^†^TB, mmol/L | 0.190 (-0.046 to 0.405) | 0.103 | -0.136 (-0.358 to 0.101) | 0.245 |
| ^†^TG, mmol/L | 0.513 (0.318-0.667) | ＜0.001 | -0.411 (-0.588 to -0.196) | ＜0.001 |
| ^†^TC, mmol/L | 0.054 (-0.182 to 0.284) | 0.648 | -0.084 (-0.311 to 0.153) | 0.475 |
| ^†^HDL-C, mmol/L | -0.290 (-0.491 to -0.061) | 0.011 | 0.235 (0.002-0.444) | 0.042 |
| ^†^LDL-C, mmol/L | 0.117 (-0.120 to 0.341) | 0.318 | -0.192 (-0.407 to 0.043) | 0.099 |
| ^†^HbA1c, % | 0.313 (0.086-0.509) | 0.006 | -0.233 (-0.442 to 0.000) | 0.044 |
| ^†^LSM, kPa | -0.206 (-0.420 to 0.028) | 0.076 | 0.275 (0.044-0.477) | 0.017 |

Abbreviations: CHB, Chronic viral hepatitis B; ALD: Alcoholic liver disease; MASLD: metabolic dysfunction-associated steatotic liver disease; BMI, body mass index; SCD, skin-to-capsulate distance; WBC, white blood cell; PLT, platelet count; AST, aspartate aminotransferase; ALT, alanine aminotransferase; ALP, alkaline phosphatase; γ-GGT, γ-glutamyl transpeptidase; TB, total bilirubin; TG, triglycerides; TC, total cholesterol; HDL-C, high-density lipoprotein cholesterol; LDL-C, low-density lipoprotein cholesterol; HbA1c, haemoglobinA1c; CT(L/S), CT liver-to-spleen attenuation ratio; UDFF, ultrasound-derived fat fraction; LSM, liver stiffness measurement.

Notes: *indicates categorical variables, analyzed using Pearson's correlation coefficient, ^†^indicates continuous variables, used Spearman's correlation coefficient. All results are reported as *r*.

**Table S4** Multicollinearity Diagnostics for UDFF and CT(L/S) Multivariate Linear Regression Model

| Variable | UDFF | | CT(L/S) | |
| --- | --- | --- | --- | --- |
|  | Tolerance | VIF | Tolerance | VIF |
| CHB | 0.278 | 3.594 | 0.253 | 3.958 |
| MASLD | 0.340 | 2.940 | 0.273 | 3.668 |
| Age | 0.550 | 1.819 | 0.529 | 1.892 |
| BMI | 0.253 | 3.955 | 0.249 | 4.010 |
| SCD | 0.314 | 3.189 | 0.309 | 3.233 |
| ALT | 0.952 | 1.050 | 0.297 | 3.364 |
| γ-GGT | 0.551 | 1.815 | 0.548 | 1.823 |
| HDL-C | 0.861 | 1.162 | 0.857 | 1.167 |
| HbA1c | 0.732 | 1.366 | 0.730 | 1.370 |
| TG | 0.479 | 2.089 | 0.474 | 2.110 |
| LSM |  |  | 0.694 | 1.440 |
| AST |  |  | 0.289 | 3.446 |
| CT scanner |  |  | 0.905 | 1.105 |

Abbreviations: CHB, Chronic viral hepatitis B; MASLD: metabolic dysfunction-associated steatotic liver disease; BMI, body mass index; SCD, skin-to-capsulate distance; ALT, alanine aminotransferase; γ-GGT, γ-glutamyl transpeptidase; HDL-C, high-density lipoprotein cholesterol; HbA1c, haemoglobinA1c; TG, triglycerides; LSM, liver stiffness measurement. AST, aspartate aminotransferase; CT(L/S), CT liver-to-spleen attenuation ratio; UDFF, ultrasound-derived fat fraction.

Notes: VIF, variance inflation factors. VIF value＜5 no significant multicollinearity, 5＜VIF value ＜10 Moderate collinearity, VIF value＞10 Severe collinearity


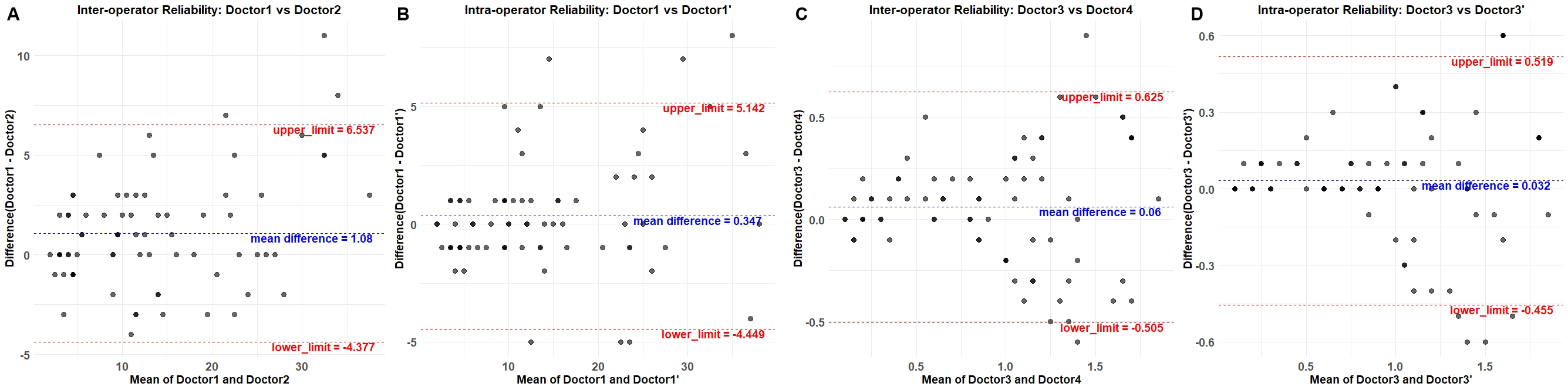


**FIGURE S1**  **Reliability analysis.** (A, C) Bland-Altman plots of inter-operator agreement (Doctor1 vs. Doctor2 for UDFF; Doctor3 vs. Doctor4 for CT[L/S]). (B, D) Bland-Altman plots of intra‑operator agreement (Doctor1 for UDFF and Doctor3 for CT[L/S]). Doctor1' and Doctor3' represent the UDFF and CT(L/S) values repeatedly measured by Doctor1 and Doctor3, respectively. UDFF, ultrasound-derived fat fraction; LSM, liver stiffness measurement; CT(L/S), CT liver-to-spleen attenuation ratio.


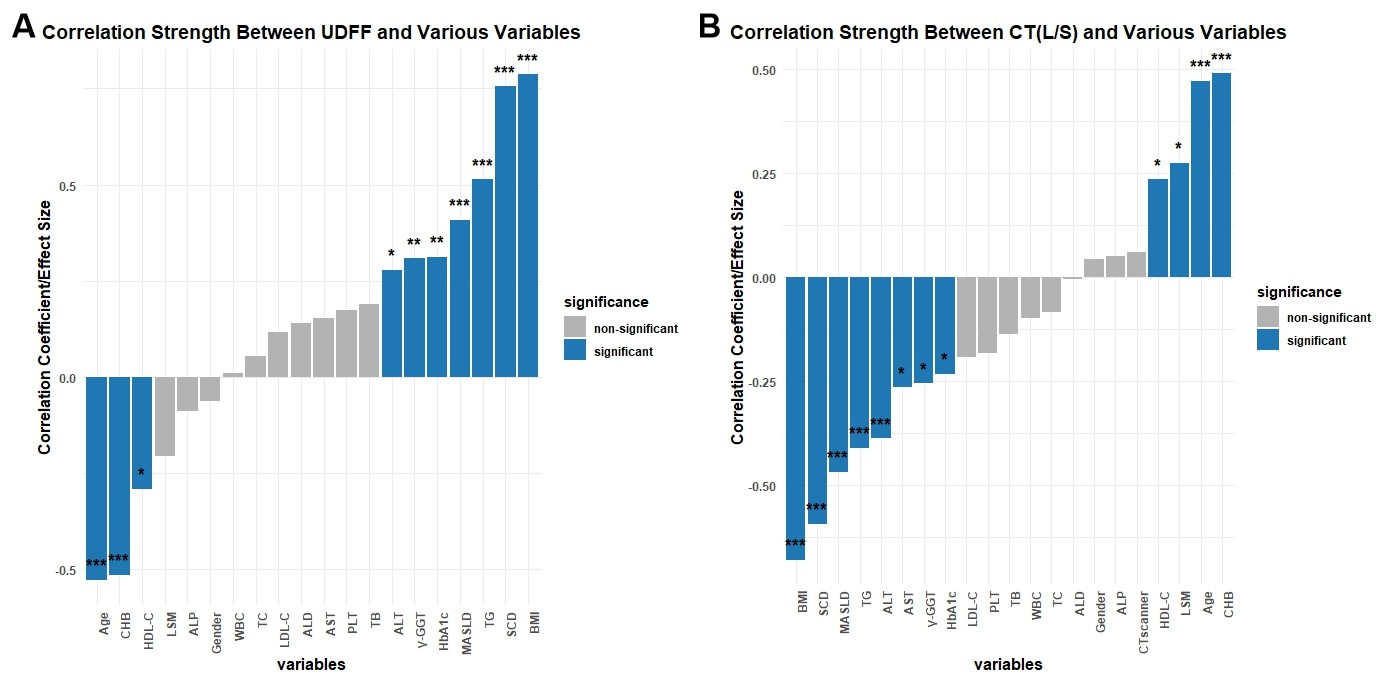


**FIGURE S2** Bar charts showing Spearman's (for continuous variables) or Pearson's (for categorical variables) correlation coefficients between UDFF (A) and CT(L/S) (B) and clinical parameters. Blue bars indicate significant correlations; grey bars indicate non-significant ones. Significance levels: * *P* < 0.05, ** *P* < 0.01, ****P* < 0.001. UDFF, ultrasound-derived fat fraction; CT(L/S), CT liver-to-spleen attenuation ratio.
